# Oncolytic reovirus preferentially induces apoptosis in *KRAS* mutant colorectal cancer cells, and synergizes with irinotecan

**DOI:** 10.18632/oncotarget.1921

**Published:** 2014-04-24

**Authors:** Radhashree Maitra, Raviraja Seetharam, Lydia Tesfa, Titto A. Augustine, Lidija Klampfer, Matthew C. Coffey, John M. Mariadason, Sanjay Goel

**Affiliations:** ^1^ Department of Oncology, Montefiore Medical Center, Bronx, NY; ^2^ Albert Einstein College of Medicine, Bronx, NY; ^3^ Oncolytics Inc., Calgary, Canada; ^4^ Ludwig Institute for Cancer Research, Melbourne, Australia; ^5^ Southern Research Institute, Birmingham, AL

**Keywords:** Colorectal cancer, reovirus, apoptosis, p21, caspase3

## Abstract

Reovirus is a double stranded RNA virus, with an intrinsic preference for replication in *KRAS* mutant cells. As 45% of human colorectal cancers (CRC) harbor *KRAS* mutations, we sought to investigate its efficacy in *KRAS* mutant CRC cells, and examine its impact in combination with the topoisimerase-1 inhibitor, irinotecan. Reovirus efficacy was examined in the *KRAS* mutant HCT116, and the isogenic *KRAS* WT Hke3 cell line, and in the non-malignant rat intestinal epithelial cell line. Apoptosis was determined by flow cytometry and TUNEL staining. Combination treatment with reovirus and irintoecan was investigated in 15 CRC cell lines, including the HCT116 p21 isogenic cell lines. Reovirus preferentially induced apoptosis in *KRAS* mutant HCT116 cells compared to its isogenic *KRAS* WT derivative, and in *KRAS* mutant IEC cells. Reovirus showed a greater degree of caspase 3 activation with PARP 1 cleavage, and preferential inhibition of p21 protein expression in *KRAS* mutant cells. Reovirus synergistically induced growth inhibition when combined with irinotecan. This synergy was lost upon p21 gene knock out. Reovirus preferentially induces apoptosis in *KRAS* mutant colon cancer cells. Reovirus and irinotecan combination therapy is synergistic, p21 mediated, and represents a novel potential treatment for patients with CRC.

## INTRODUCTION

Colorectal cancer (CRC) is the second leading cause of cancer mortality in the US, accounting for around 50,000 deaths annually [1]. The key genes and signaling pathways which drive CRC formation have been extensively described [2], and include the WNT, RAS-MAPK, PI3K, TGF-β and P53 pathways. While surgically curable if detected early, 5 year survival rates for patients with inoperable metastatic disease is less that 10%. Hence novel treatments for this disease are urgently needed.

The use of oncolytic viruses as a treatment for cancer has been increasingly explored over the last decade [(3)]. In particular, these organisms have been evaluated as anti-tumor agents due to their ability to selectively replicate in cells with activation of specific oncogenes [4]. Mammalian reovirus is a ubiquitous non enveloped double stranded (ds) RNA virus normally associated with relatively benign pathology in humans. The Dearing strain of reovirus serotype 3 (ReoT3D) is a non-engineered wild type reovirus strain with innate ability to kill *KRAS* transformed cells [5]. This was directly demonstrated in NIH 3T3 cells, where conditional expression of mutant *KRAS* promoted productive viral replication [4, 6]. The association of dsRNA dependent protein kinase (PKR) and effective reoviral replication is well established [7]. PKR dimerization, autophosphorylation, and activation, upon binding to dsRNA are the critical step towards prohibiting viral translation initiation in *KRAS* wild type cells. Specific chemical inhibitors of PKR phosphorylation lead to enhancement of reovirus translation in untransformed cells [7].

Several studies have attempted to elucidate the precise mechanism of reovirus induced oncolysis. It has been reported that reoviral oncolysis is beta interferon independent and is enhanced by interferon regulatory factor 3 and NF-κB-dependent expression of Noxa, a protein that promotes activation of caspases and apoptosis [8]. Activation of caspase 3 has also been reported to be necessary for development of reovirus induced encephalitis [9]. On the contrary, a recent study reported that reovirus exerts potent apoptotic effects in head and neck cancer cell lines in a caspase 3 independent manner [10].

Reovirus is being actively clinically investigated as a novel cancer therapy with 13 trials completed and 18 trials ongoing in various cancers [11]. The virus has been therapeutically tested in over 300 patients both intratumorally (ITu) and intravenously (IV), and both, as a monotherapy or in combination with radiotherapy or chemotherapy in multiple tumor types including head and neck, colon, lung, and pancreas.

Activating mutations in *KRAS* occur in approximately 40-45% of patients with CRC [10]. Recent clinical data demonstrates that the anti-EGFR antibodies, cetuximab and panitumumab, are ineffective in patients with CRC whose tumors harbor *KRAS* mutations [12]. New treatments are therefore particularly needed for this patient subgroup. While reovirus has demonstrated increased oncolytic activity in *KRAS* activated cells, the efficacy of the virus has not been comprehensively tested in colon cancer cells.

In the current study we demonstrate preferential reoviral oncolysis in *KRAS* mutant CRC cell lines. This effect is associated with activation of caspase 3 and PARP-1 cleavage, along with the repression of p21 protein. Furthermore, we demonstrate that the combination treatment of reovirus and irinotecan synergistically induced growth arrest and apoptosis in colon cancer cells, in a p21 dependent manner.

## RESULTS

### Reovirus preferentially induces growth inhibition in KRAS mutant cells

**Figure 1a:**
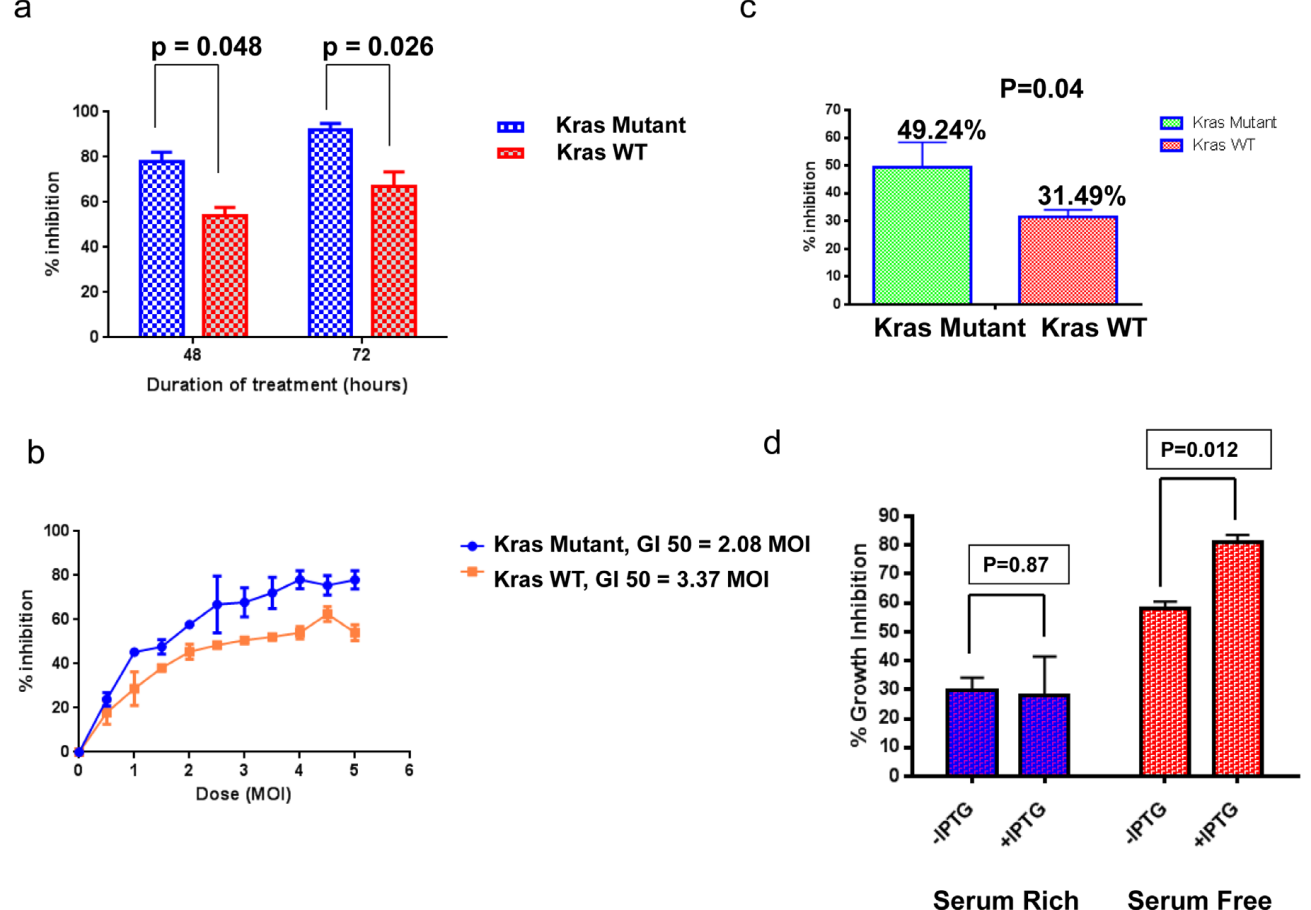
Effect of reovirus on the KRAS isogenic cell lines. There was a preferential sensitivity to reovirus in the *KRAS* mutant HCT116 cell line as compared to the *KRAS* WT Hke3 cell line. At 48 hours, the mean + SEM growth inhibition was 78.08% (+ 4.11%) for the *KRAS* mutant cell line vs. 54.14% (+ 3.59%) for the *KRAS* WT cell line, with a p value of 0.048. Similarly, at 72 hours, the mean (+ SEM) growth inhibition was 91.78% (3.08%) for the *KRAS* mutant cell line as compared to 67.12% (6.32%) for the *KRAS* WT cell line, with a p value of 0.026. **b** Effect of reovirus at range of doses between MOI 0.5-5 in *KRAS* mutant HCT116 and *KRAS* WT Hke3 cell lines. Data presented as % inhibition (mean + SEM) at each of the doses. A regression curve was ascertained and using the curve so generated, the GI50 was derived to be 2.08 MOI for *KRAS* mutant HCT116 and 3.37 MOI for Hke3. **c**. Growth inhibition patterns in a panel of 13 CRC cell lines. Reovirus induced greater (49.24 + 9.09%) growth inhibition in 5 *KRAS* mutated, than the 8 *KRAS* wild type (31.49 + 2.6%) cell lines at 72 hours at a dose of 2.5 MOI with significant p value of 0.04. All values reported as mean + SEM.**d**. MTT assay confirms reovirus activity under KRAS induced and un-induced condition confirming greater activity under KRAS induced situation. Reovirus induces greater growth inhibition in IEC-iKRAS rat epithelial cell line and the inhibition is significant (p= 0.012) in the serum free environment.

The effect of reovirus on growth inhibition was examined in *KRAS* mutant HCT116 cells and its *KRAS* wild type isogenic derivative Hke 3 using the MTT assay. We saw no activity at the 24 hour time point with the HCT116 cell line, and this was not pursued for the other cell lines. We observed a preferential sensitivity to reovirus in the *KRAS* mutant HCT116 cell line as compared to the *KRAS* WT Hke3 cell line, as shown in figure 1a. At 48 hours, the mean + Standard Error of Mean (SEM) growth inhibition was 78.08% (+ 4.11%) for the *KRAS* mutant cell line vs. 54.14% (+ 3.59%) for the *KRAS* WT cell line, with a p value of 0.048. Similarly, at 72 hours, the mean (+ SEM) growth inhibition was 91.78% (+ 3.08%) for the *KRAS* mutant cell line as compared to 67.12% (+ 6.32%) for the *KRAS* WT cell line, with a p value of 0.026. We then analyzed the effect of using various concentrations of reovirus on the two cell lines to enable calculation of growth inhibition of 50% of cells (GI50). Reovirus was studied at concentrations ranging from 0.5 to 5 MOI, and a regression curve was created. Using the curve so derived, the GI50 was calculated to be 2.08 MOI for *KRAS* mutant HCT116 and 3.37 MOI for the *KRAS* WT Hke3 (Figure 1b).

We next extended and verified our findings in an extended panel of an additional 11 CRC cell lines comprising a total of 5 *KRAS* mutant and 8 *KRAS* WT lines (Figure 1c). On average, reovirus inhibited cell growth by 49.24 ± 9.09% (mean +/- SEM) in *KRAS* mutant cell lines, and by 31.49 ± 2.62% (mean +/- SEM) in *KRAS* WT lines (P = 0.04). We factored in the potentially differential growth rates between the 2 cell lines by subtracting the absorbance at time 0 from control and treatment groups. Furthermore, we did not find any difference in the control values of both cell lines at the 48 and 72 hour time point, further adding to the confidence that the differential effects were only attributable to the differential susceptibility of the cell lines, rather than the potentially differential growth rates.

Finally, this analysis was extended to the non-transformed rat intestinal epithelial cell (IEC)-iKRAS epithelial cell line with inducible *KRAS* under the control of lac operon. Mutant *KRAS* was induced by treatment with 5 mM IPTG for 72h prior to reovirus infection. While no significant difference in reovirus induced growth inhibition was observed between control (29.74 ± 4.43%) and mutant *KRAS* expressing cells (27.82 ± 3.59%) (mean +/- SEM) when grown under normal serum condition, a significant difference was observed when cells were cultured the cells in serum free media (58.15% ± 2.31% inhibition vs. 80.80% ± 2.7% inhibition (mean +/- SEM) for control and cells expressing mutant *KRAS*, respectively; P=0.012, Figure 1d).

To confirm preferential sensitivity to reovirus as dependent of the *KRAS* status of the cell line, we analyzed sensitivity of the isogenic cells to irinotecan and did not observe any difference in sensitivity to irinotecan (p=0.66, supplementary figure 1).

### Reovirus infection results in S phase reduction, G2/M arrest and cell membrane disruption in CRC cells

**Figure 2ai:**
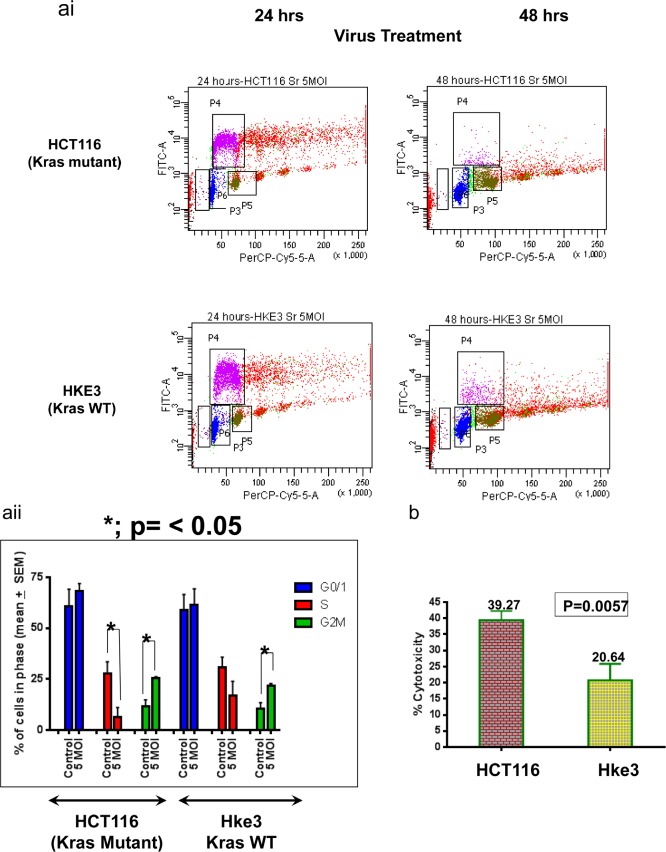
Flow cytometric analysis of HCT116 and Hke3 at 24 and 48 hours of treatment. The FITC labeled BrdU incorporated cells indicates the S phase population which decreases with increase of time. The effect is most pronounced for *KRAS* mutated HCT116 cells. **aii**. A graphical representation of the cell cycle distribution upon treatment with 5 MOI reovirus for 48 hours. 10,000 events were recorded and compared between treated and untreated population. In HCT116 (*KRAS* mutant) cells, there is a prominent ablation of S-phase population upon reovirus infection (P= 0.008), but not for *KRAS* WT cells. A significant G2M arrest was observed in both the cell lines (p= 0.0133 for HCT116, and p=0.028 for Hke3). **b**. LDH cytotoxicity assay as a measurement of cell membrane destabilization post reovirus infection. Cells were treated for 72 hours at 2MOI and LDH released to the culture media was quantified. The percent cytotoxicity was significantly higher (39.27% ± 2.9%) for HCT116 as compared to (20.64%± 5.2%) for Hke3 (p= 0.0057). All values are mean + SEM.

To investigate the mechanism by which reovirus induced growth inhibition of colon cancer cells, we examined the effect of reovirus infection on cell cycle kinetics. The *KRAS* mutant HCT116 and *KRAS* WT Hke3 cells were infected with reovirus 5MOI and harvested at 48 hours post infection, with treatment with 10uM BrdU 1 hour prior to harvest. Cells were stained with a FITC-conjugated anti BrdU antibody and propidium iodide and the distribution of cells in the various phases of the cell cycle determined by FACS analysis. A significant G2M arrest was observed in both the cell lines. In the KRAS mutant HCT116 cell line, the G2M phase increased from 11.68% + 3.23% to 25.47% + 0.67%, mean + SEM (p= 0.0133). In the KRAS WT Hke3 cell line, the G2M phase increased from 10.38% + 3.12% to 21.58% + 1.28%, mean + SEM (p= 0.028). Consistent with the MTT data, reovirus led to S phase ablation preferentially in the *KRAS* mutant HCT116 cell line (from 27.60% + 3.42 to 6.28 + 2.78%, p=0.008) as compared the *KRAS* WT Hke3 cells (from 30.74% to 17.00%, p=0.184) (Figure 2ai and ii).

To determine if reovirus infection induces membrane disruption, we examined the effect of reovirus treatment on release of the cytosolic protein lactate dehydrogenase (LDH) into the culture medium. Reovirus infection at a MOI of 2 for 72 hours induced significantly higher release of LDH in *KRAS* mutant HCT116 cells (39.27 ± 2.9%) compared to *KRAS* WT Hke3 cells (20.64 ± 5.2%; p=0.0057) (Figure 2b), indicating greater disruption of membrane integrity in *KRAS* mutant HCT116 cells. All values are mean + SEM.

### Reovirus utilizes the extrinsic apoptotic pathway with Caspase 3 activation

**Figure 3a:**
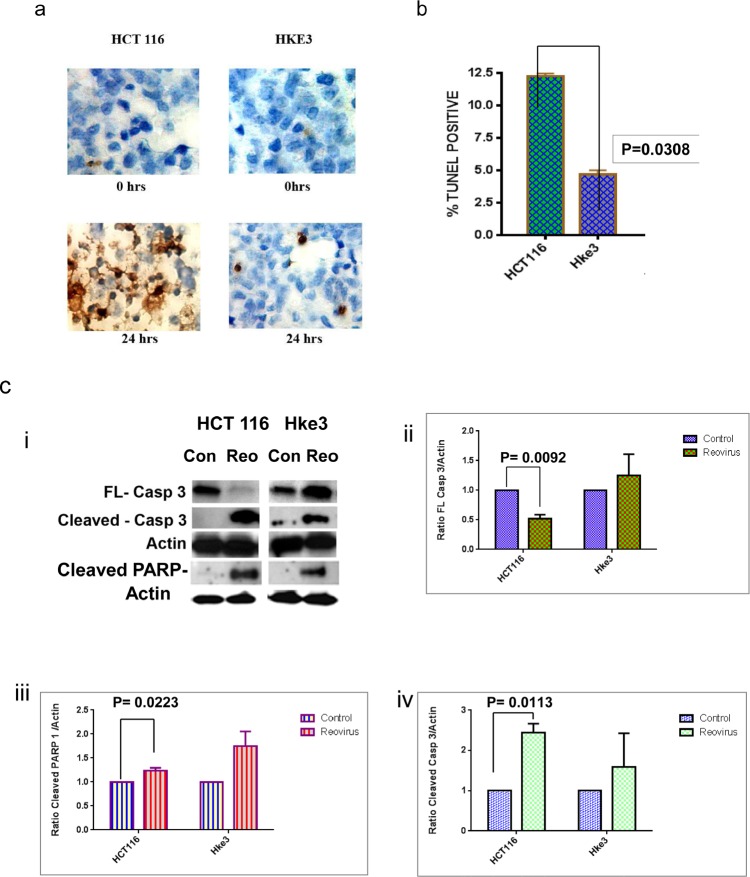
A microscopic photograph of TUNEL stained cells at 0 and 24hrs post treatment. The brown stain represents the apoptotic cells which are higher for *KRAS* mutated HCT116 cells. **b**. A graphical representation of the prevalence of TUNEL positive apoptotic cells at 24 hours post reovirus treatment in HCT116 and Hke3 cells. The graph shows the mean from two independent experiment with 12.22 ±0.24% (mean ± SEM) apoptotic TUNEL positive cells in HCT116 and Hke3 cells 4.66 ± 0.345% (mean ±SEM) cells in Hke3. A two tail t test is employed to generate the p value of 0.03.**c**. i.Photographic representation of expression of full length and cleaved caspase 3 proteins, and cleaved PARP-1 in control and reovirus treated HCT116 and Hke3 cells as quantified by western blot analysis. The cells were treated at 5MOI and harvested at 48 hours post treatment. 60 ugm of protein was loaded in each lane to quantitatively follow the expression of the proteins. A prominent cleavage of full length caspase 3 along with enhanced cleaved PARP-1 is noted in reovirus treated HCT116 cells. ii-iv. The adjoining graphs represent the relative densitometry of the three proteins normalized to ß-Actin in control and reovirus treated HCT116 and Hke3 cells. The effect of down regulation of full length caspase 3 and up regulation of cleaved PARP-1 and cleaved caspase 3 is significant in reovirus treated *KRAS* mutant HCT116 cells only. The graph shows the mean protein densities from two independent experiments and a two tailed t test is employed to generate the p value.

To determine whether reovirus infection induces apoptosis in colon cancer cells, we performed TUNEL staining pre and post reovirus treatment. Cells were grown on slides and fixed for staining 24 hours post virus infection. Reovirus infection induced a significantly higher percentage of TUNEL positive cells in *KRAS* mutant HCT116 cells with 12.22 ±0.24% (mean ± SEM) apoptotic TUNEL positive cells compared to Hke3 cells 4.66 ± 0.345% (mean ±SEM) (Figure 3a and b). The difference was noted to be significant with a p value of 0.03.

We next performed western blot analysis to explore the molecular events that drive the enhanced apoptosis of *KRAS* mutant HCT116 cells following reovirus infection. The cleavage of caspase 3 (pro–form) was significantly higher in HCT116 cells along with increased levels of cleaved PARP-1 at 48 hours post virus treatment (5MOI) when compared to Hke3 (Figure 3ci). The relative densitometry (normalized to β-Actin) of full length caspase 3 (p=0.0092) (figure 3cii), cleaved PARP-I (p=0.0223) (figure 3ciii) and cleaved caspase 3 (p=0.0113) (figure 3civ) was observed as a significant difference in the *KRAS* mutant HCT116 cells only, and not in the *KRAS* WT Hke3 cells.

### Reovirus infection causes a greater degree of viral crystalline array in KRAS mutant colon cancer

**Figure 4a:**
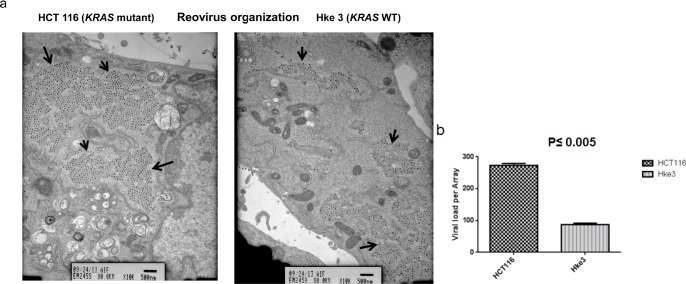
Transmission electron micrograph of HCT116 and Hke3 cells upon 5MOI reovirus treatment for 48 hours. The *KRAS* mutant HCT116 cells show a much greater formation of viral crystalline Arrays (VCA's) indicative of the effective generation of infection competent virion particles. The images were photographed under 10K magnification. **b**. Graphical representation of the number of viral particles per array in HCT116 and Hke3 cells treated with 5MOI reovirus at for 48 hours. Three independent arrays were counted per cell line. A quantitative analysis of count of viral particle per array indicated 272.67 ± 3.71 (mean ± SEM) particles in HCT116 and 87±2.65 (mean ± SEM) particles in Hke3 cells. A significant p value of p≤0.005 was observed.

The TEM images clearly shows a higher number of viral crystalline arrays (VCA) formation in *KRAS* mutant HCT 116 cells as compared to *KRAS* WT Hke3 cells at 48 hours (Figure 4a). A quantitative analysis of count of viral particle per array in the two cell lines showed 272.67 ± 3.71 (mean ± SEM) particles in HCT116 while it was lower in the *KRAS* WT Hke 3 cells with 87± 2.65 (mean ± SEM) particles with a significant difference (p ≤ 0.005) (Figure 4b). These data are consistent with the previous findings of HCT116 cells being more susceptible to the reovirus as compared to Hke 3 cells. The array formation is displayed in the cytoplasm and no preferential sub cellular organelle localization is observed in either of the cell lines.

### Combination treatment with reovirus and the topoisomerase I inhibitor irinotecan synergistically inhibits cell growth of colorectal cancer cell lines

**Figure 5a:**
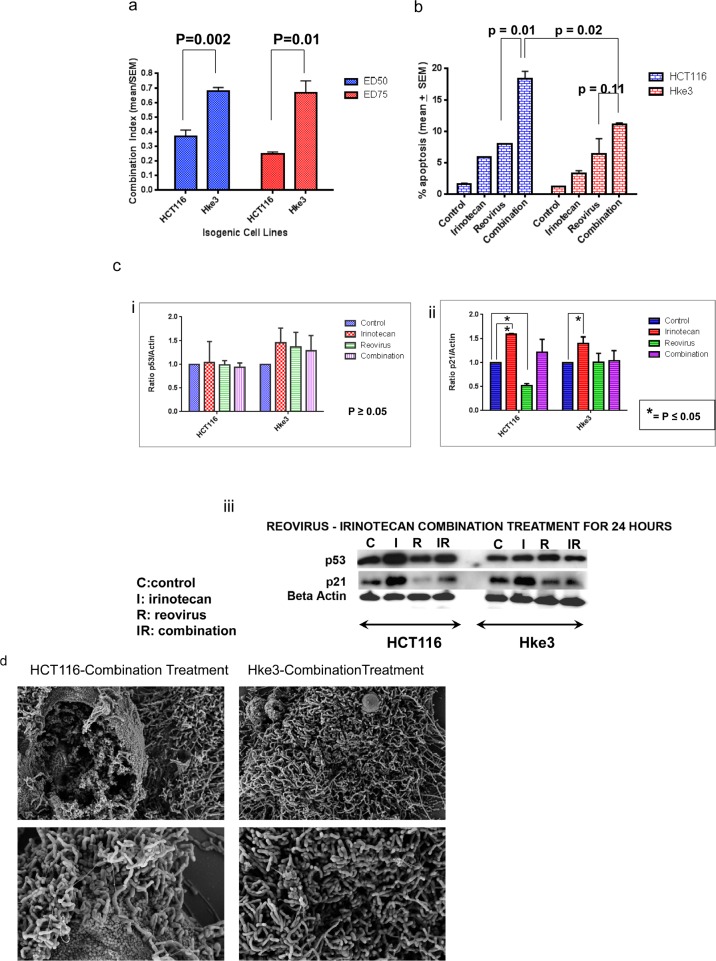
Combination index of reovirus and irinotecan administration in KRAS mutant HCT116 as compared to KRAS wild type isogenic Hke3 cells at 50% and 75% growth inhibition. While synergy is observed in both cell lines (CI<1), a significant difference is detected in the CI between the two cell lines at the respective effective doses (ED; p=0.002 at ED50 and p=0.01at ED75 respectively).**b**. FACS analysis for quantitative assessment of apoptosis. The combination group showed a greater degree of apoptosis than single agent reovirus (p=0.01) in HCT116, while in the KRAS WT Hke3 cells there was no improvement as compared to single agent reovirus (p=0.11; Figure 5b). Moreover, the apoptosis in the combination was significantly higher in the KRAS mutant cells at 18.44 + 1.07 (mean + SEM) than the KRAS WT cells, at 11.14 + 0.16 (mean + SEM), with a p value of 0.02. **c**. Western blot assay to determine the expression of p21 and p53 proteins. HCT116 and Hke3 cells were treated with 5MOI reovirus and 2 uM irinotecan as single agent and in combination for 24 hours. Cells were harvested and 50 ugm were loaded per lane. The blot was probed with β-actin to confirm the equal protein load per lane. The adjoining graphs represents the relative densitometry of p53 and p21 proteins normalized to ß-Actin in control, irinotecan, reovirus and combination treatment of HCT116 and Hke3 cells at 48 hours. The expression of p53 was not significant in either of the cell lines but p21 showed a significant upregulation in irinotecan treated groups in both the cell lines where as significant downregulation only in reovirus treated *KRAS* mutant HCT 116 cells. The graph shows the mean protein densities from two independent experiments and a two tailed t test is employed to generate the p value. **d**. Scanning electron micrograph of HCT116 and Hke3 cells upon combination treatment at 5K magnification (upper panel) and 10K magnification in the lower panel. More prominent perturbation is observed in *KRAS* mutant HCT116 when compared to *KRAS* wildtype Hke3 cells.

As the clinical advancement of this agent as a treatment option for patients with colon cancer may require its combination with standard treatments, we examined the effect of reovirus when administered in combination with the topoisomerase I inhibitor, irinotecan. First, we compared the effect of the combination at a concentration of 1 MOI reovirus and 1uM irinotecan, in *KRAS* mutant HCT116 and KRAS WT Hke3 cells on cell growth after 48 hours treatment. In HCT116 cells, reovirus and irinotecan alone inhibited cell growth by 54.48 + 2.73% and 8.80 + 2.78% respectively, while the combination inhibited cell growth by 79.50 + 1.44% (mean +/- SEM). Similarly, in Hke3 cells, reovirus and irinotecan alone inhibited cell growth by 42.31 + 4.01% and 10.37 + 3.37% respectively, while the combination inhibited cell growth by 58.16 + 4.19% (mean +/- SEM). To determine if the effect of the combination was synergistic; we computed the combination index using the calcusyn program. To do so, we expanded the experiments to test the combination at concentrations ranging from 0.5 to 10 MOI (for reovirus) and 0.5 to 10 uM (for irinotecan), alone as single agents, and in combination. For both HCT116 and Hke3 cells, the combination induced a synergistic inhibition of cell growth as indicated by the combination index (CI) of <1. Interestingly, the CI was lower at both ED 50 (*KRAS* mutant 0.37 + 0.04; *KRAS* WT 0.68 + 0.02; mean +SEM; p=0.002) and ED 75 (*KRAS* mutant 0.25 + 0.01; *KRAS* WT 0.67 + 0.08; mean +/- SEM; p=0.01) for the *KRAS* mutant HCT116 than for the *KRAS* WT Hke3 cell lines (Figure 5a).

To confirm these findings using an independent assay, we next examined the effect of this combination on apoptosis after 48 hours treatment (Figure 5b). In HCT116 cell lines, apoptosis was induced by 3.05 fold following single agent irinotecan treatment, 5.05 fold by reovirus treatment and 11.05 fold by the combination. In Hke3 cells: apoptosis was induced by 2.79 fold following single agent irinotecan treatment, 5.47 fold by reovirus treatment and 9.58 fold by the combination. The combination group showed a greater degree of apoptosis than single agent reovirus (p=0.01) in *KRAS* mutant HCT116, while in the *KRAS* WT Hke3 cells there was no improvement as compared to single agent reovirus (p=0.11; Figure 5b). Moreover, the apoptosis in the combination was significantly higher in the KRAS mutant cells at 18.44 + 1.07 (mean + SEM) than the KRAS WT cells, at 11.14 + 0.16 (mean + SEM), with a p value of 0.02.

To determine the mechanistic basis for this synergy we examined the effect of each agent, alone and in combination on induction of p53 and its target gene, p21. As expected, irinotecan induced p53 with a parallel induction of p21 expression in HCT116 cells. In comparison, reovirus had minimal effect on p53 induction (Figure 5c i & iii), but surprisingly repressed p21 expression (Figure 5c ii & iii). In the combination arm, p21 levels were similar to that in the control. One possibility therefore is that the lack of induction of p21 may tip the balance between growth arrest and apoptosis that occurs following p53 induction in favor of apoptosis, resulting in a synergistic enhancement of apoptosis.

SEM images show a greater extent of perturbation of the cell surface morphology in HCT116 as compared to Hke3 (Figure 5d and supplementary Figure 2) upon 48 hours of combination treatment, further confirming the previous findings that the *KRAS* mutant HCT 116 cell line is a better candidate for irinotecan and reovirus mediated synergistic cell cytotoxicity.

Finally, we examined the effect of this combination on the extended CRC cell line panel. Combining reovirus with irinotecan synergistically induced cytotoxicity in 12/13 cell lines (Table 1). As observed in the HCT116 isogenic system, the synergistic effect of the combination was observed in both *KRAS* WT and mutant cell lines.

**Table 1:** The combination indices of a panel of CRC cell lines as calculated by calcusyn software The combination index (CI) was calculated and interpreted as follows: CI < 1 = Synergy; 1 = Additive effects; >1 = Antagonism. Data is presented at as CI value at 50% and 75% effective dose. Among the 13 CRC cell lines, 8 were KRAS WT and 5 were KRAS mutant. Twelve cell lines showed synergy upon combination treatment, while one namely SW948 showed antagonism [1, 34].

	Cell Line	ED50	ED75	Kras status
1	SW403	0.00019	0.00134	MUTANT
2	Colo 201	0.18693	0.23786	WT
3	KM12	0.26191	0.16842	WT
4	HCT116	0.36829	0.24973	MUTANT
5	HCT 15	0.38077	0.23928	MUTANT
6	RW 2982	0.41196	0.14481	WT
7	RKO	0.43368	0.51396	WT
8	Caco 2	0.4431	0.29098	WT
9	HCT 8	0.47913	0.35206	MUTANT
10	HT 29	0.53254	0.49114	WT
11	SW620	0.55425	0.38694	MUTANT
12	Hke3	0.68212	0.67016	WT
13	SW948	1.12079	1.4288	WT

### The synergistic cytotoxicity of irinotecan and reovirus is p21 dependent

To demonstrate the central role of p21 mediating the synergistic effect of reovirus and irinotecan, we repeated the combination experiments in the isogenic partners, HCT 116 p21 +/+ and HCT 116 p21 −/−, using the MTT assay. The knockdown of p21 protein expression was verified by western blot analysis (Supplementary figure 3). Most interestingly, the synergy observed with the combination was retained in the HCT 116 p21 +/+ cells (CI < 1) but was lost in the HCT 116 p21 −/− cells, and was in fact suggestive of antagonism (CI > 1) (Table 2, and supplementary figures 4 and 5).

**Table 2:** Combination index of the HCT 116 isogenic cell lines, namely p21 ^+/+^ and p21 ^−/-^ were studied Synergy was observed in the former, while antagonism was seen in the latter.

	Cell Line	ED50	ED75
1	HCT116 p21 +/+	0.56315	0.64849
2	HCT116 p21 −/−	2.46 × 103	6.19 × 104

## DISCUSSION

Metastatic CRC remains an incurable illness with a median survival time of approximately 2 years. A sub group of colon cancer patients (~40%) harbor mutations in the *KRAS* oncogene, and are precluded from receiving anti-EGFR targeted therapies [13]. The lack of alternate treatments for these patients makes this an area of urgent investigation and unmet medical need. The preferential oncolysis of *KRAS* mutant cancer cells by reovirus [7] prompted us to investigate this as a potential therapeutic option for these patients. Moreover, while the safety, feasibility and potential efficacy of reovirus as a cancer therapy are currently being evaluated in multiple phase I-III clinical trials, the underlying molecular mechanism of by which reovirus preferentially induces oncolysis in *KRAS* mutant cells is not well understood.

While prior reports using model systems of mouse NIH-3T3 cells and glioblastoma cell lines [6, 7, 14] have demonstrated reovirus induced oncolysis, the current study is the most comprehensive evaluation of the susceptibility of CRC cell lines to reovirus. First, we utilized an isogenic model system, using the parental HCT116 cells (with mutant *KRAS*) and its isogenic derivative, Hke3 cells (*KRAS* WT), wherein the mutant *KRAS* allele has been deleted by homologous recombination [15]. This was associated with a more pronounced decrease in the percentage of cells in S phase, a significant G2M arrest, a more pronounced release of LDH. The decrease in S phase upon treatment with reovirus is consistent with previous data, however, in that study, there was a G1 arrest with little change in G2M, unlike our observation. This is partially explicable by the difference in cell lines, as we used only CRC cells, while in the other, exclusively head and neck cancer cells were used [16]. Second, we demonstrated that the *KRAS* mutation also facilitates reovirus susceptibility in a non-malignant model. Interestingly, the sensitivity of mutant *KRAS* expressing cells was only evident under serum free conditions indicating that the presence of an exogenous factor(s) may dampen the influence of *KRAS* mutation on reovirus sensitivity, or compensates for the lack of a *KRAS* mutation. Although beyond the scope of this manuscript, the identification of this factor(s) that can physiologically compensate for mutant *KRAS* may provide valuable insight into how response to reovirus is determined. Furthermore, using a panel of 13 CRC cell lines, we demonstrated increased susceptibility of *KRAS* mutant cells to the reovirus therapy, as compared to the *KRAS* WT cells.

Our findings shed light on the mechanism by which reovirus induces cytotoxicity in colon cancer cells. The observed induction of apoptosis in a caspase 3 dependent manner was notably more pronounced in *KRAS* mutant HCT 116 cells. These findings are consistent with a previous study where caspase-3 activation was required for reovirus induced encephalitis *in vivo*, with caspase-3 (−/−) mice showing lesser degree of tissue damage with better survival [9], but contradictory to another report that suggests that reovirus mediated cell death is independent of caspase activation, but rather, is mediated by a process called necroptosis, an alternate necrotic form of cell death [17]. It therefore appears that reovirus mediates cell death in multiple ways and remains a topic of further study.

Reovirus has limited single agent efficacy, and its therapeutic potential will likely be realized in combination therapies. The use of synergistically acting drug combinations for treating cancer can lower the doses of each constituent drug and consequently lower adverse effects [18]. Irinotecan, a topoisomerase I inhibitor that leads to DNA replication arrest and DNA damage [19] is a potent cytotoxic agent and commonly used as second line chemotherapy for patients with mCRC. Evaluation of the efficacy of reovirus and irinotecan aims in identifying a plausible therapy targeting the oxaliplatin refractory CRC patients. It is plausible that reovirus will find applicability when combined with oxaliplatin and 5-FU, and will likely be the topic of future studies that we hope to perform. The combination synergistically induced growth inhibition and apoptosis in multiple cell lines. At the molecular level, irinotecan strongly induced p21 expression in HCT116 cells, consistent with prior reports [20, 21]; while conversely, reovirus suppressed p21 expression. As p21 has been shown to be able to protect cells from stress-induced apoptosis [22], the inhibition of irinotecan induced p21 expression may provide an explanation for the synergistic effect of the combination. These findings are also consistent with those reported previously by Zhang et al where caspase-mediated repression/inactivation of p21 converts cancer cells from a growth arrested to an apoptotic state [21]. Finally to establish the proposed contribution of p21 towards the synergistic effect of irinotecan and reovirus combination we analyzed the drug effects in p21 +/+ and p21 −/− (knock out) HCT116 CRC cells. When synergy was computed by calcusyn software the p21 +/+ cells clearly demonstrated synergy, while the p21 −/− lacked synergy, instead demonstrating antagonism. Although a major effect of p21, a cyclin-dependent kinase inhibitor, is considered to be exerted during G1 phase of the cell cycle, p21 gene knock-out studies suggested its involvement in G2/M checkpoint as well [23].

Our flow cytometry analysis showed a clear G2M arrest with prominent S-phase ablation upon reovirus treatment, the effect being significantly pronounced under *KRAS* mutant conditions. The p21−/− cells showed a lower degree reovirus infectivity and loss of synergy upon irinotecan combination suggesting that p21 plays a crucial role in inducing double drug mediated cellular cytotoxicity.

Finally, our observation that reovirus synergistically induces apoptosis when combined with irinotecan is consistent with previous studies in which this agent has been combined with other chemotherapeutics. These include combination with paclitaxel, docetaxel, and gemcitabine in various models [3, 16, 24, 25]. Furthermore, reovirus has previously been shown to be synergistic with radiation by enhancement of apoptosis, wherein doses of radiation upto 5 Gy and reovirus to 1 MOI were investigated [26]. These findings have led to the successful implementation of human trials in the appropriate indication, such as with gemcitabine in pancreatic cancer, and with carboplatin/paclitaxel in head and neck cancer[11].

Reovirus has demonstrated safety as a single agent across multiple phase I studies including one at our institution [27]. Our current findings that reovirus is preferentially active in KRAS mutant CRC, and its observed synergy with irinotecan, has prompted the initiation of a phase I trial testing the reovirus with FOLFIRI (folinic acid, 5-FU, and irinotecan; a standard second line option for patients with metastatic CRC) [28] in KRAS mutant mCRC, and preliminary results have been encouraging, with median progression free survival (PFS) of 7.4 months [29]. This is comparable to the data reported in two recent trials, with PFS of 5.7 and 6.9 months when FOLFIRI was combined with anti VEGF agents, bevacizumab [30] and aflibercept [31], respectively. Combination of traditional cytotoxic therapies with such novel biological agents as reovirus gives hope to the future of therapeutic improvement for patients with KRAS mutant mCRC.

## METHODS

### Cell lines and growth conditions

The CRC cell lines SW403, Colo201, KM12, HCT116, HCT15, RW2982, RKO, Caco2, HCT8, HT29, SW620 and SW948 were obtained from the ATCC. The *KRAS* mutant HCT116 cell line and its isogenic derivative, Hke3 in which mutant *KRAS* has been deleted by homologous recombination have been previously described [15] was kindly provided by Dr. S.Shirasawa to Dr. L Klampfer. Non transformed mutant *KRAS* inducible rat intestinal epithelial cells (IEC-I *Kras*) were kindly provided by Dr.Raymond DuBois [32]. This cell line expresses *Kras*^Val12^ under the control of the lac operon, and is induced by treatment with 5 mM Isopropyl β-D-1-thiogalactopyranoside (IPTG, Life Technologies, Inc., Gaithersburg, MD) [32]. The HCT116 p21−/− cell line along with the parental HCT 116 p21 +/+ was kindly provided by Prof. Bert Vogelstein. All cell lines were cultured in MEM (Gibco BRL), supplemented with 10% FBS, 2mM L-Glutamine and 1% penicillin streptomycin. For serum free experiments the cell culture media PC-1 without L- Gln was used (Lonza #77232).

### Reovirus infection and irinotecan treatment

Reovirus type 3 dearing strain (trade name Reolysin^®^) was provided by Oncolytics Biotech Inc. (Calgary, Canada) at a TCID_50_ of 2.53 x10^10^ particles per ml concentration. Virus particles were stored in the dark at −80°C for long term storage and at +4°C for 4 weeks. Appropriate dilutions were performed in growth media immediately prior to initiation of infection. Cells were infected for 6 hours followed by a change of media and infected cells were grown for a further 24-72 hours at 37°C. The chemotherapy drug, irinotecan (chemical name, (S)-4,11-diethyl-3,4,12,14-tetrahydro-4-hydroxy-3,14-dioxo1Hpyrano[3',4':6,7]-indolizino[1,2-b]quinolin-9-yl-[1,4'bipiperidine]-1'-carboxylate, monohydrochloride, trihydrate; empirical formula C33H38N4O6•HCl•3H2O) was obtained from the Montefiore Medical Center oncology outpatient pharmacy as Camptosar at a concentration of 20 mg/ml and diluted in culture media to the desired final concentration at the time of treatment.

### MTT assay for cell proliferation

For determination of reovirus sensitivity, 5,000 to 10,000 cells per well were seeded in 96-well plates and treated with reovirus at 0, 0.5, 1, 2, 5 or 10 multiplicity of infection [31] [31] for 24, 48 and 72 hours. For each cell line, one plate was harvested at the time of viral infection for determination of t = 0 absorbance values. Viable cells were determined post treatment using the 3-(4,5-dimethylthiazol-2-yl)-2,5-diphenyltetrazolium bromide (MTT) (Sigma M2128) assay by measurement of absorbance at 570 nm [33]. The relative rate of cell growth for each cell line was factored into the analysis by subtracting the absorbance at time 0 from both the control and treatment groups. For calculation of the GI50 of reovirus, KRAS isogenic cell lines were treated with reovirus at concentrations ranging from 0.5 to 5 MOI and readings taken at 48 hours. All experiments were repeated at least three times.

### Fluorescence activated cell sorting analysis - Cell cycle distribution

For assessment of the effect of reovirus on cell cycle distribution and apoptosis, cells were treated with 10uM BRDU 1 hour prior to harvesting. Cells were washed, treated with FITC conjugated mouse anti-BrdU antibody (BD Pharmingen #556028) for 1 hour, and stained with 50 μg/mL propidium iodide (Sigma P4170) for 30 minutes at room temperature. Fluorescence-activated cell sorting (FACS) analysis was performed as previously described [33] and the raw data analyzed using modfit 3.2.1 software.

### TUNEL staining

The paraffin embedded reovirus treated and untreated HCT116 and Hke3 cells packed in histogel were deparaffinized and hydrated by transferring them through the following solutions: twice in xylene for 5 min, twice in 96% ethanol, 90% ethanol, 80% ethanol, and finally double distilled water (DDW), for 3 minutes. Nuclei were stripped of proteins by incubation with 20 pg/ml proteinase K (PK; Sigma Chemical) for 15 minutes at room temperature, following which the slides were washed in distilled water for 2 minutes. Endogenous peroxidase was inactivated by incubation with 2% H_2_O_2_ for 5 minutes at room temperature. The sections were rinsed with distilled water and immersed in terminal deoxynucleotidyl transferase (TdT) buffer (30 mM Trizma base, pH 7.2, 140 mM sodium cacodylate, 1mM cobalt chloride, Boehringer Mannheim, Mannheim, Germany) followed by the addition of biotinylated deoxyuridine triphosphate (dUTP) (Boehringer Mannheim). dUTP was diluted in TdT buffer at a concentration of 0.15 (Endotoxin Unit) EU/ml. The solution was placed on the sections, and then incubated in a humidified chamber at 37°C for 60 minutes. The reaction was terminated by transferring the slides to TB buffer (300 mM sodium chloride, 30 mM sodium citrate) for 15 min at room temperature. The sections were rinsed with DDW, covered with 2% human serum albumin for 10 minutes at room temperature, rinsed again in DDW, and immersed in PBS for 5 minutes. The sections were covered with Streptavidin Peroxidase (Dako, Santa Barbara, CA) diluted 1:100 in PBS, incubated for 30 min at 37°C, washed three times in PBS and stained with 3,3'-diaminobenzidineas a substrate for the peroxidase for approximately 30 min at 37°C. Counter staining was performed using Mayer's hematoxylin. A quantification of the number of TUNEL positive cells in a given visual field were enumerated by manual counting when cells were viewed under 40X magnification.

### Electron Microscopy

Transmission Electron Microscopy (TEM)

Monolayer cell cultures of HCT116 and Hke3 [control, irinotecan (2μM), reovirus (5MOI) and combination] were fixed with 2% para-formaldehyde, 2.5% glutaraldehyde, in 0.1 M sodium cacodylate buffer pH 7.4 at 4 °C, postfixed with 1% osmium tetroxide followed by 2% uranyl acetate, dehydrated through a graded series of ethanol, cells lifted from the monolayer with propylene oxide and embedded as a loose pellet in LX112 resin (LADD Research Industries, Burlington VT) in eppendorf tubes. Ultrathin sections were cut on a Reichert Ultracut UCT, (0.5μm). Semi-thin sections were stained with 1:1 mixture of 1.0% methylene blue and 1.0% Azure B, observed with a light microscope, and subsequently selected regions were thin-sectioned and collected on 300 mesh copper grids. The grids were finally stained with uranyl acetate followed by lead citrate and viewed on a JEOL 1200EX transmission electron microscope at 80kv under 10K magnification.

### Scanning Electron Microscopy (SEM):

HCT116 and Hke3 [control, irinotecan (2μM), reovirus (5MOI) and combination] cells were fixed in 2.5% glutaraldehyde, 0.1 M sodium Cacodylate, 0.2 M Sucrose, 5mM MgCl2 pH 7.4 and dehydrated through a graded series of ethanol, then critically point dried using liquid carbon dioxide in a Tousimis Samdri 795 Critical Point Drier (Rockville MD). They were next sputter coated with chromium in a Quorum EMS 150T ES (Quorum Technologies Ltd, United Kingdom) and examined in a Zeiss Supra Field Emission Scanning Electron Microscope (Carl Zeiss Microscopy, LLC North America), using an accelerating voltage of 3KV and observed at 1K, 5K and 10K magnification.

### LDH Assay

Cell death and lysis (cytotoxicity) was assessed by measurement of lactate dehydrogenase (LDH) levels in the medium using the LDH release cytotoxicity detection kit (ABCAM #65393) according to manufacturer's instructions. All samples were measured at an absorbance of 450 nm using a micro titer plate reader.

### Western Blot Analysis

Western blots were performed using standard procedures. Membranes were blocked with 5% milk in TBS containing 0.1% Tween 20, and incubated with antibodies specific for p21, p53 and caspase 3 (Santa Cruz biotechnologies: Sc 6246, Sc55476, Sc7272 respectively), cleaved caspase 3, and cleaved PARP-1 (Cell signaling technologies # 9661S and # 5625P) and β-actin (A3853, Sigma Aldrich, St. Louis, MO). Immunoreactive bands were visualized by chemiluminescence (RPN 2232, Amersham ECL western blotting detection kit, Piscataway, NJ). Relative densitometric values (expression of protein of interest normalized to ß-Actin) were determined using Image J Software (NIH) and represents the mean of two blots from two independent experiments.

### Statistical Analysis

Cytotoxicity experiments were performed at least 3 times and the mean values from different treatment groups compared by two way unpaired student's t test with a p value < 0.05 considered statistically significant. The dose effect analysis of single and combination treatments was performed using software CalcuSyn Version 2.0. The software is a definitive analyzer of combined drug effects, able to compute synergism and antagonism using the median effect method described by Chou and Talalay [34]. The combination index (CI) was calculated and interpreted as follows: CI < 1 = synergy; 1 = additive effects; >1 = antagonism. The data is presented as CI value at effective dose. The GI50 for the isogenic cell lines was determined by generation of regression curves of growth inhibition (mean + SEM) when treated with reovirus at doses between MOI of 0.5-5 at 48 hours treatment, using the Graphpad Prism v 6.0 software program (Graphpad Software, San Diego, CA)

## SUPPLEMENTARY FIGURES



## References

[1] Siegel R, DeSantis C, Virgo K, Stein K, Mariotto A, Smith T, Cooper D, Gansler T, Lerro C, Fedewa S, Lin C, Leach C, Cannady RS, Cho H, Scoppa S, Hachey M (2012). Cancer treatment and survivorship statistics, 2012. CA Cancer J Clin.

[2] East JE, Dekker E (2013). Colorectal cancer diagnosis in 2012: A new focus for CRC prevention-more serration, less inflammation. Nat Rev Gastroenterol Hepatol.

[3] Sei S, Mussio JK, Yang QE, Nagashima K, Parchment RE, Coffey MC, Shoemaker RH, Tomaszewski JE (2009). Synergistic antitumor activity of oncolytic reovirus and chemotherapeutic agents in non-small cell lung cancer cells. Mol Cancer.

[4] Shmulevitz M, Marcato P, Lee PW (2005). Unshackling the links between reovirus oncolysis, Ras signaling, translational control and cancer. Oncogene.

[5] Marcato P, Shmulevitz M, Lee PW (2005). Connecting reovirus oncolysis and Ras signaling. Cell Cycle.

[6] Strong JE, Coffey MC, Tang D, Sabinin P, Lee PW (1998). The molecular basis of viral oncolysis: usurpation of the Ras signaling pathway by reovirus. EMBO J.

[7] Coffey MC, Strong JE, Forsyth PA, Lee PW (1998). Reovirus therapy of tumors with activated Ras pathway. Science.

[8] Knowlton JJ, Dermody TS, Holm GH (2012). Apoptosis induced by mammalian reovirus is beta interferon (IFN) independent and enhanced by IFN regulatory factor 3- and NF-kappaB-dependent expression of Noxa. J Virol.

[9] Beckham JD, Tuttle KD, Tyler KL (2010). Caspase-3 activation is required for reovirus-induced encephalitis in vivo. J Neurovirol.

[10] Twigger K, Roulstone V, Kyula J, Karapanagiotou EM, Syrigos KN, Morgan R, White C, Bhide S, Nuovo G, Coffey M, Thompson B, Jebar A, Errington F, Melcher AA, Vile RG, Pandha HS (2012). Reovirus exerts potent oncolytic effects in head and neck cancer cell lines that are independent of signalling in the EGFR pathway. BMC Cancer.

[11] Maitra R, Ghalib MH, Goel S (2012). Reovirus: a targeted therapeutic--progress and potential. Mol Cancer Res.

[12] Lievre A, Bachet JB, Le Corre D, Boige V, Landi B, Emile JF, Cote JF, Tomasic G, Penna C, Ducreux M, Rougier P, Penault-Llorca F, Laurent-Puig P (2006). KRAS mutation status is predictive of response to cetuximab therapy in colorectal cancer. Cancer Res.

[13] Lievre A, Bachet JB, Boige V, Cayre A, Le Corre D, Buc E, Ychou M, Bouche O, Landi B, Louvet C, Andre T, Bibeau F, Diebold MD, Rougier P, Ducreux M, Tomasic G (2008). KRAS mutations as an independent prognostic factor in patients with advanced colorectal cancer treated with cetuximab. J Clin Oncol.

[14] Norman KL, Lee PW (2000). Reovirus as a novel oncolytic agent. J Clin Invest.

[15] Shirasawa S, Furuse M, Yokoyama N, Sasazuki T (1993). Altered growth of human colon cancer cell lines disrupted at activated Ki-ras. Science.

[16] Roulstone V, Twigger K, Zaidi S, Pencavel T, Kyula JN, White C, McLaughlin M, Seth R, Karapanagiotou EM, Mansfield D, Coffey M, Nuovo G, Vile RG, Pandha HS, Melcher AA, Harrington KJ (2012). Synergistic cytotoxicity of oncolytic reovirus in combination with cisplatin-paclitaxel doublet chemotherapy. Gene Ther.

[17] Berger AK, Danthi P (2013). Reovirus activates a caspase-independent cell death pathway. MBio.

[18] Gravitz L (2011). Chemoprevention: First line of defence. Nature.

[19] O'Dwyer PJ, Catalano RB (2006). Uridine diphosphate glucuronosyltransferase (UGT) 1A1 and irinotecan: practical pharmacogenomics arrives in cancer therapy. J Clin Oncol.

[20] Motwani M, Jung C, Sirotnak FM, She Y, Shah MA, Gonen M, Schwartz GK (2001). Augmentation of apoptosis and tumor regression by flavopiridol in the presence of CPT-11 in Hct116 colon cancer monolayers and xenografts. Clin Cancer Res.

[21] Zhang Y, Fujita N, Tsuruo T (1999). Caspase-mediated cleavage of p21Waf1/Cip1 converts cancer cells from growth arrest to undergoing apoptosis. Oncogene.

[22] Gartel AL, Tyner AL (2002). The role of the cyclin-dependent kinase inhibitor p21 in apoptosis. Mol Cancer Ther.

[23] Ando T, Kawabe T, Ohara H, Ducommun B, Itoh M, Okamoto T (2001). Involvement of the interaction between p21 and proliferating cell nuclear antigen for the maintenance of G2/M arrest after DNA damage. J Biol Chem.

[24] Pandha HS, Heinemann L, Simpson GR, Melcher A, Prestwich R, Errington F, Coffey M, Harrington KJ, Morgan R (2009). Synergistic effects of oncolytic reovirus and cisplatin chemotherapy in murine malignant melanoma. Clin Cancer Res.

[25] Heinemann L, Simpson GR, Boxall A, Kottke T, Relph KL, Vile R, Melcher A, Prestwich R, Harrington KJ, Morgan R, Pandha HS (2011). Synergistic effects of oncolytic reovirus and docetaxel chemotherapy in prostate cancer. BMC Cancer.

[26] Twigger K, Vidal L, White CL, De Bono JS, Bhide S, Coffey M, Thompson B, Vile RG, Heinemann L, Pandha HS, Errington F, Melcher AA, Harrington KJ (2008). Enhanced in vitro and in vivo cytotoxicity of combined reovirus and radiotherapy. Clin Cancer Res.

[27] Gollamudi R, Ghalib MH, Desai KK, Chaudhary I, Wong B, Einstein M, Coffey M, Gill GM, Mettinger K, Mariadason JM, Mani S, Goel S (2010). Intravenous administration of Reolysin, a live replication competent RNA virus is safe in patients with advanced solid tumors. Invest New Drugs.

[28] http://clinicaltrials.gov/ct2/show/NCT01274624?term=reovirus+irinotecan&rank=1.

[29] Allyson J, Ocean TSB-S, Imran Chaudhary, Romae Palmer, Paul J. Christos, Alice Mercado, Erika O. Florendo, Veronica A. Rosales, Joseph T. Ruggiero, Elizabeta C. Popa, Melissa Wilson, Mohammad Haroon Ghalib, Yijuan Hou, Umang Shah, Lakshmi Rajdev, Tarek Elrafei, George M. Gill, Matthew C. Coffey, Manish A. Shah, Sanjay Goel (2012). A multicenter phase I study of intravenous administration of reolysin in combination with irinotecan/fluorouracil/leucovorin (FOLFIRI) in patients (pts) with oxaliplatin-refractory/intolerant KRAS-mutant metastatic colorectal cancer (mCRC). J Clin Oncol.

[30] Bennouna J, Sastre J, Arnold D, Osterlund P, Greil R, Van Cutsem E, von Moos R, Vieitez JM, Bouche O, Borg C, Steffens CC, Alonso-Orduna V, Schlichting C, Reyes-Rivera I, Bendahmane B, Andre T (2013). Continuation of bevacizumab after first progression in metastatic colorectal cancer (ML18147): a randomised phase 3 trial. Lancet Oncol.

[31] Van Cutsem E, Tabernero J, Lakomy R, Prenen H, Prausova J, Macarulla T, Ruff P, van Hazel GA, Moiseyenko V, Ferry D, McKendrick J, Polikoff J, Tellier A, Castan R, Allegra C (2012). Addition of aflibercept to fluorouracil, leucovorin, and irinotecan improves survival in a phase III randomized trial in patients with metastatic colorectal cancer previously treated with an oxaliplatin-based regimen. J Clin Oncol.

[32] Sheng H, Shao J, Dubois RN (2001). K-Ras-mediated increase in cyclooxygenase 2 mRNA stability involves activation of the protein kinase B1. Cancer Res.

[33] Mariadason JM, Rickard KL, Barkla DH, Augenlicht LH, Gibson PR (2000). Divergent phenotypic patterns and commitment to apoptosis of Caco-2 cells during spontaneous and butyrate-induced differentiation. J Cell Physiol.

[34] Chou TC, Talalay P (1984). Quantitative analysis of dose-effect relationships: the combined effects of multiple drugs or enzyme inhibitors. Adv Enzyme Regul.

