# Supplementary material for: Oncolytic reovirus preferentially induces apoptosis in KRAS mutant colorectal cancer cells, and synergizes with irinotecan
Source: Oncotarget. 2014 Apr 24;5(9):2807–19. doi: 10.18632/oncotarget.1921 (PMC4058046; doi:10.18632/oncotarget.1921)
Supplement: Supplementary file 1 [file oncotarget-05-2807-s001.pdf]

Oncolytic reovirus preferentially induces apoptosis in KRAS mutant colorectal cancer cells, and synergizes with irinotecan

Supplementary Figures

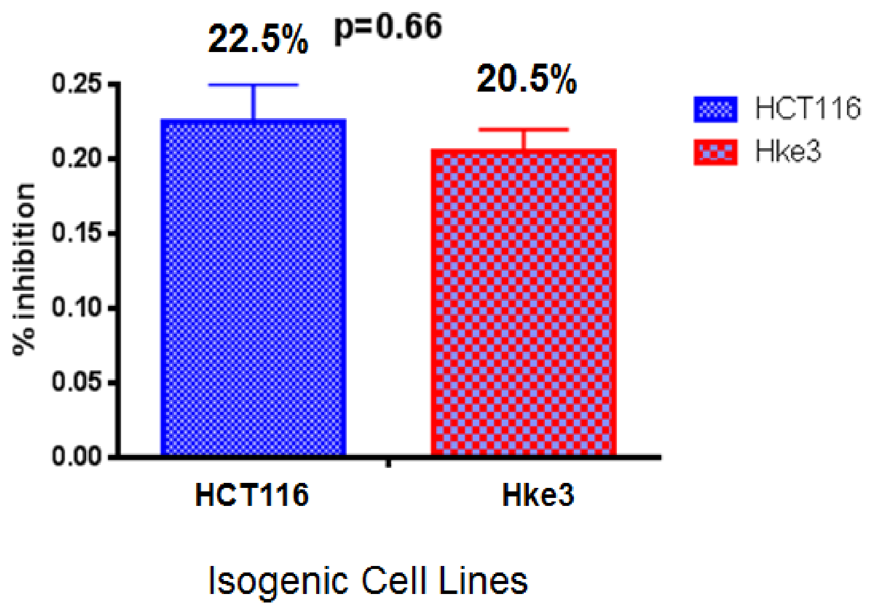

Supplementary Figure 1: A graphical representation of 50% inhibitory effect of 2.5 uM irinotecan on HCT116 and Hke3 at 72 hours. There is no significant growth inhibition between the two cell lines (p=0.66)

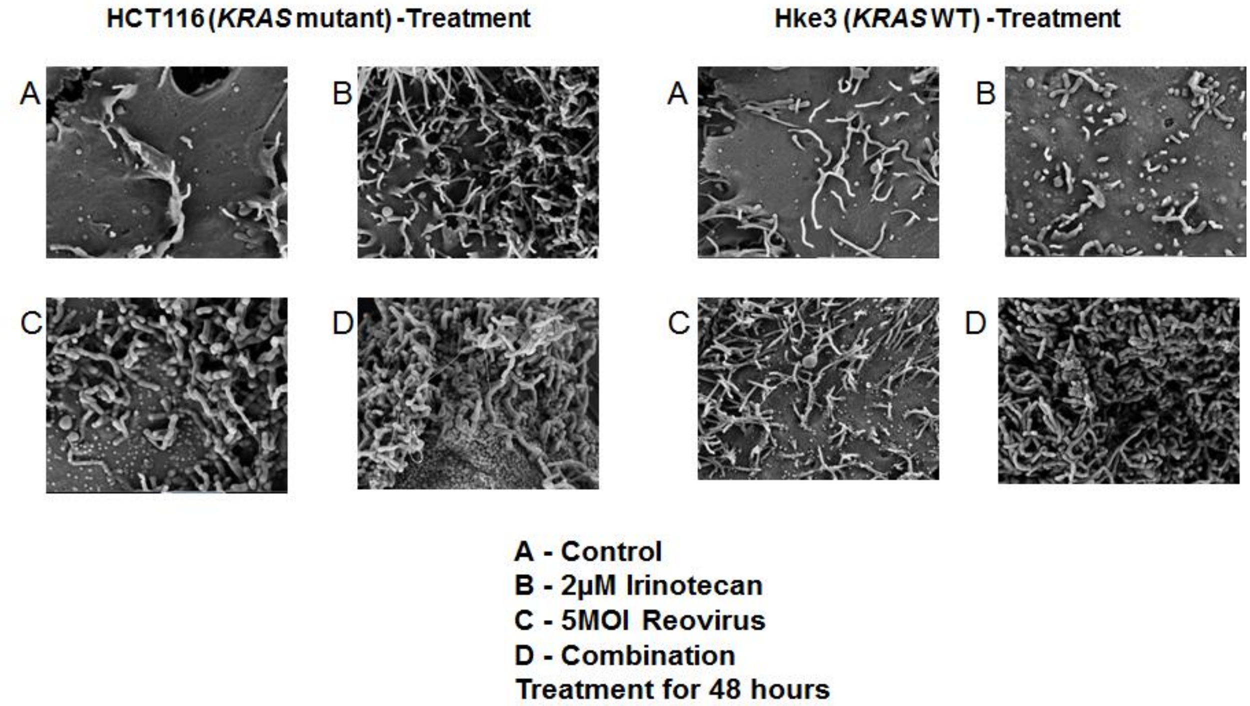

Supplementary Figure 2: Scanning electron micrograph of HCT116 and Hke3 cells for all 4 treatment condition namely control, irinotecan (2μM), reovirus (5MOI) and combination as observed under 10K magnification using SE2 Lens. We see a more prominent surface structure alteration in Reovirus treated cells and the combination in HCT116.

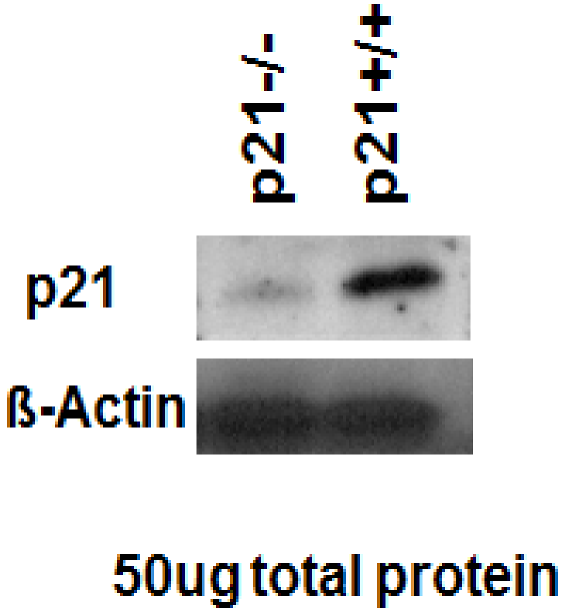

Supplementary Figure 3: Western Blot probed with anti-p21 mouse monoclonal antibody for total cellular protein extract from HCT116 p21+/+ and HCT116 p21-/- cells. 50 µgm of protein were loaded in each lane. Equal loading was confirmed by probing for ß actin housekeeping gene product. HCT116 p21-/- showed a clear down regulation of p21 expression.

Median Effect Plots

A: HCT 116 p21 +/+

B: HCT 116 p21 -/-

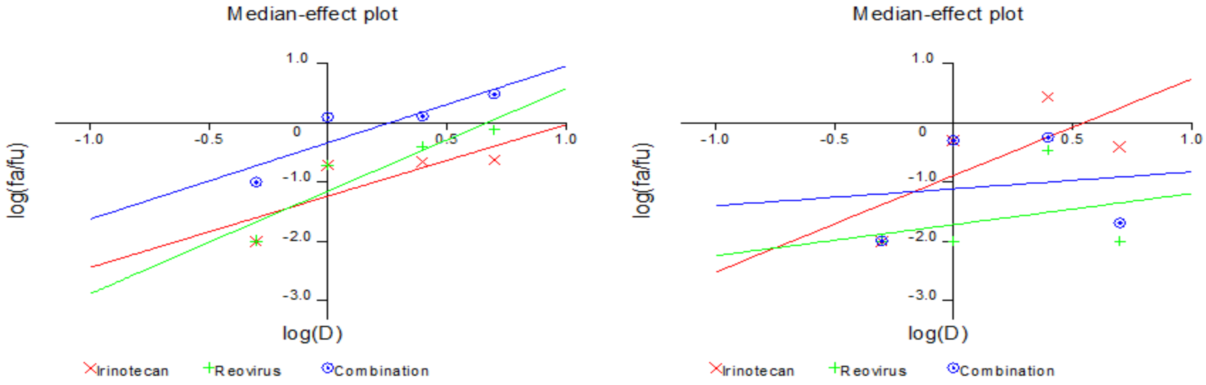

Supplementary Figure 4: Median effect plots (MEP) of the combination of irinotecan and reovirus in isogenic HCT 116 p21 cell lines. In “A”, is shown the MEP of the p21 expressing cell line showing synergistic action, while in “B” is the p21 null cell line depicting antagonism. (Please also refer to table 2b, that depicts the combination indices).

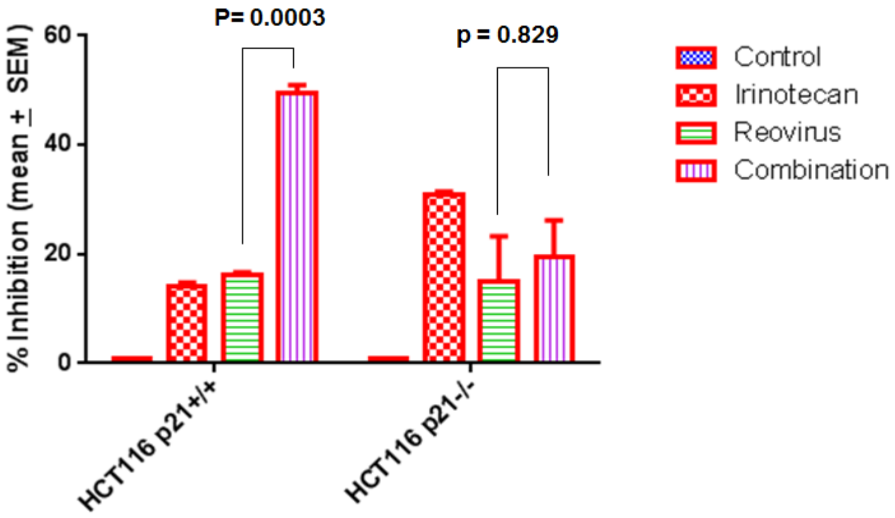

Supplementary Figure 5: Graphical representation of the percent inhibition of reovirus and irinotecan as single agent and in combination on p21-/- and p21+/+ HCT116 cells at 48 hrs. A clear amplification of growth inhibition was observed in the parental HCT116 cells upon combination treatment which was absent in p21-/- under identical treatment conditions.
